# Supplementary material for: Evaluation of the Determination of Dabigatran, Rivaroxaban, and Apixaban in Lupus Anticoagulant-Positive Patients
Source: Diagnostics (Basel). 2021 Nov 2;11(11):2027. doi: 10.3390/diagnostics11112027 (PMC8623477; doi:10.3390/diagnostics11112027)
Supplement: Supplementary file 1 [file diagnostics-11-02027-s001.zip › diagnostics-1405396-supplementary.pdf]

diagnostics

Article

Evaluation of the Determination of Dabigatran, Rivaroxaban, and Apixaban in Lupus Anticoagulant-Positive Patients

Jana Úlehlová [1,†], Barbora Piskláková [2,†], Eliška Ivanovová [2], Jana Procházková [1], Pavla Bradáčová [1], Aleš Kvasnička [2], David Friedecký [2*], Luděk Slavík [1*]

[1] Department of Hematology-Oncology, Faculty of Medicine and Dentistry, Palacký University and University Hospital Olomouc, 779 00 Olomouc, Czech Republic; jana.ulehlova@fnol.cz (J.Ú.); jana.prochazkova@fnol.cz (J.P.); pavla.bradacova@kzcz.eu (P.B.)
[2] Laboratory for Inherited Metabolic Disorders, Department of Clinical Biochemistry, Palacký University and University Hospital Olomouc, 779 00 Olomouc, Czech Republic; barbora.pisklakova@upol.cz (B.P.); eliska.ivanovova@gmail.com (E.I.); ales.kvasnicka01@upol.cz (A.K.)
* Correspondence: david.friedecky@upol.cz (D.F.); Tel.: +420585852619; ludek.slavik@fnol.cz (L.S.); Tel.: +420585855350
† These authors contributed equally to this work.

Citation: Úlehlová, J.; Piskláková, B.; Ivanovová, E.; Procházková, J.; Bradáčová, P.; Kvasnička, A.; Friedecký, D.; Slavík, L. Evaluation of the Determination of Dabigatran, Rivaroxaban, and Apixaban in Lupus Anticoagulant-Positive Patients. *Diagnostics* **2021**, *11*, 2027. https://doi.org/10.3390/diagnostics11112027

Academic Editor: Claudia Maria Radu

Received: 22 September 2021
Accepted: 27 October 2021
Published: 2 November 2021

Publisher's Note: MDPI stays neutral with regard to jurisdictional claims in published maps and institutional affiliations.

Copyright: © 2021 by the authors. Licensee MDPI, Basel, Switzerland. This article is an open access article distributed under the terms and conditions of the Creative Commons Attribution (CC BY) license (http://creativecommons.org/licenses/by/4.0/).

Table S1. Lupus LSR levels and concentrations of direct anticoagulant levels evaluated by functional tests or by HPLC-MS/MS method before and after DOAC Stop procedure. All concentration values are in units ng/ml.

Patient	Lupus anticoagulants (LSR)	DABI_FA	DABI_LC-MS	RIVA_FA	RIVA_LC-MS	API_FA	API_LC-MS	DABI_FA_DS	DABI_LC-MS_DS	RIVA_FA_DS	RIVA_LC-MS_DS	API_FA_DS	API_LC-MS_DS
P1	2.19	110.80	30.37	109.90	31.90	104.50	346.30	76.70	0.12	88.80	0.73	96.00	21.86
P2	1.36	103.30	25.47	91.30	34.26	107.10	499.30	63.80	0.44	62.10	0.10	100.90	34.74
P3	2.13	168.00	50.25	154.00	46.91	144.20	269.30	111.40	0.39	113.30	0.67	123.80	23.05
P4	1.78	90.40	37.82	101.20	38.12	72.90	373.90	64.80	0.32	74.50	0.42	74.00	9.75
P5	1.51	76.70	32.41	77.40	40.14	74.80	246.80	50.20	0.22	46.60	0.10	78.80	2.13
P6	1.63	68.00	32.49	61.20	49.89	72.20	215.70	44.40	0.22	54.90	1.16	76.80	0.10
P7	2.56	90.80	27.61	87.10	49.13	113.80	212.00	68.90	0.25	79.40	0.53	82.30	3.72
P8	1.23	76.90	57.68	72.60	71.44	105.80	200.20	47.10	0.31	45.40	0.67	66.90	3.60
P9	1.96	106.00	51.19	133.10	85.17	115.10	186.80	59.10	0.19	66.30	1.01	77.50	2.40
P10	1.30	86.00	56.57	74.80	81.93	110.90	218.00	42.60	0.27	45.20	1.51	66.50	3.95
P11	1.74	106.10	58.53	88.60	83.03	100.50	218.60	61.20	0.32	61.40	1.09	70.40	3.17
P12	3.72	228.00	59.90	182.00	89.54	235.00	225.40	123.20	2.56	114.20	1.56	118.60	6.68
P13	1.66	96.90	58.47	104.00	87.77	125.40	228.00	57.90	0.32	54.50	0.36	70.70	1.86
P14	5.95	235.00	65.86	235.00	93.02	235.00	221.00	213.20	0.52	218.30	1.44	146.60	2.43
P15	1.48	73.80	55.01	69.50	91.98	106.10	227.50	52.10	0.29	51.20	0.67	67.10	4.09
P16	1.34	90.30	69.80	61.00	94.07	89.70	219.10	46.50	0.28	45.10	0.28	58.60	2.80
P17	1.87	114.80	58.79	91.30	100.20	106.50	240.60	65.80	0.78	65.50	1.32	65.70	3.83
P18	1.42	87.20	47.37	57.50	95.76	112.10	193.30	53.30	0.32	50.40	3.81	67.10	6.39
P19	2.39	119.90	59.82	107.00	52.02	95.00	317.20	74.70	0.45	71.40	0.49	92.30	13.74
P20	1.53	109.60	69.64	77.60	97.03	91.50	322.00	58.10	0.33	55.50	1.17	80.50	2.98
P21	1.46	110.10	60.36	88.20	90.06	121.00	282.60	53.60	0.28	54.10	0.37	86.50	3.15
P22	1.35	92.40	58.81	84.90	107.80	79.20	387.50	53.40	0.36	51.90	1.80	74.00	7.98
P23	2.13	137.30	51.35	150.90	88.80	118.80	206.20	86.20	0.21	86.10	0.33	74.40	13.59
P24	2.50	143.70	53.57	126.00	45.49	102.70	223.60	67.20	0.17	77.90	0.33	66.90	6.29
P25	1.57	76.80	48.16	81.60	92.84	91.10	294.30	46.40	0.16	48.80	0.70	62.60	5.16
P26	1.45	76.70	29.30	61.30	59.27	104.40	234.50	48.90	0.27	46.50	0.10	65.60	1.38
P27	1.81	98.80	21.01	99.60	67.80	114.00	158.30	59.90	0.12	58.50	0.75	68.10	1.49
P28	1.61	84.30	44.85	80.50	94.87	112.40	198.70	49.90	0.17	50.80	0.89	69.70	2.29
P29	1.29	69.70	48.80	69.40	84.12	83.60	225.40	44.80	0.26	43.50	0.72	57.90	1.77
P30	3.30	140.20	36.52	156.10	72.72	130.10	205.30	93.50	0.16	98.00	0.94	79.20	2.04
P31	1.41	73.00	40.58	70.70	76.32	70.80	232.70	45.00	0.10	45.80	0.74	57.80	1.68

Abbreviations: DABI, dabigatran; RIVA, rivaroxaban; API, apixaban; FA, functional assay; DS, DOAC-Stop procedure
